# Structure and Interdomain Interactions of a Hybrid Domain: A Disulphide-Rich Module of the Fibrillin/LTBP Superfamily of Matrix Proteins

**DOI:** 10.1016/j.str.2009.03.014

**Published:** 2009-05-13

**Authors:** Sacha A. Jensen, Sarah Iqbal, Edward D. Lowe, Christina Redfield, Penny A. Handford

**Affiliations:** 1 Department of Biochemistry, University of Oxford, Oxford OX1 3QU, UK

**Keywords:** PROTEINS, CELLBIO

## Abstract

The fibrillins and latent transforming growth factor-β binding proteins (LTBPs) form a superfamily of structurally-related proteins consisting of calcium-binding epidermal growth factor-like (cbEGF) domains interspersed with 8-cysteine-containing transforming growth factor β-binding protein-like (TB) and hybrid (hyb) domains. Fibrillins are the major components of the extracellular 10–12 nm diameter microfibrils, which mediate a variety of cell-matrix interactions. Here we present the crystal structure of a fibrillin-1 cbEGF9-hyb2-cbEGF10 fragment, solved to 1.8 Å resolution. The hybrid domain fold is similar, but not identical, to the TB domain fold seen in previous fibrillin-1 and LTBP-1 fragments. Pairwise interactions with neighboring cbEGF domains demonstrate extensive interfaces, with the hyb2-cbEGF10 interface dependent on Ca^2+^ binding. These observations provide accurate constraints for models of fibrillin organization within the 10–12 nm microfibrils and provide further molecular insights into how Ca^2+^ binding influences the intermolecular interactions and biomechanical properties of fibrillin-1.

## Introduction

The 10–12 nm fibrillin microfibrils of the extracellular matrix play important roles in both elastic and nonelastic tissues. During elastogenesis, they provide a scaffold for the deposition of tropoelastin and form the periphery of the mature elastic fiber. In nonelastic tissues, such as the ciliary zonule of the eye and at basement membranes, they have an anchoring function and provide tissues with tensile strength (Kumaratilake et al., 1989). In addition to these biomechanical functions, the microfibrils are also involved in the regulation of growth factors through the interactions of their major component, the fibrillins, with the latent transforming growth factor-β binding proteins (LTBPs) and bone morphogenetic proteins (Chaudhry et al., 2007; Gregory et al., 2005; Isogai et al., 2003; Neptune et al., 2003; Sengle et al., 2008). They also interact with a variety of other cell-matrix components. The importance of these interactions is seen in the development of diseases, such as Marfan syndrome, in which mutations in fibrillin-1 lead to a loss of structural integrity in the extracellular matrix and perturbations in transforming growth factor β (TGF-β) signaling (Ramirez and Dietz, 2007).

Although it has been known for several years that fibrillin is the main structural component of the microfibrils (Sakai et al., 1986), the precise organization of individual fibrillin molecules within the microfibrils is still controversial. When viewed by rotary shadowing electron microscopy, microfibrils appear as beaded filaments with an interbead distance of 56 nm. Several models have been proposed to explain the organization of fibrillin into microfibrils based on methods such as small-angle X-ray scattering (SAXS) and multiangle laser light scattering (Baldock et al., 2001, 2006), antibody labeling of extracted microfibrils (Kuo et al., 2007; Reinhardt et al., 1996), mapping of proteolytic cleavage sites (Kuo et al., 2007), and structure determination of fibrillin fragments by nuclear magnetic resonance (NMR) and X-ray crystallography (Knott et al., 1996; Lee et al., 2004; Smallridge et al., 2003). These models can be placed into two broad groups: those in which fibrillin monomers are “folded” to fit within one microfibril interbead distance (Baldock et al., 2001, 2006) and staggered models with extended fibrillin monomers (Kuo et al., 2007; Lee et al., 2004).

**Figure 1:**
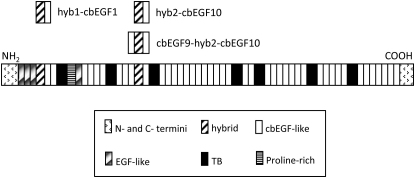
Domain Organization of Human Fibrillin-1 The fibrillin sequence is largely dominated by cbEGF domains, interspersed with TB and hyb domains. Constructs used to determine Ca^2+^ affinities of hyb-cbEGF pairs and the cbEGF9-hyb2-cbEGF10 construct are shown above the figure.

The molecular organization of the fibrillins, and the related LTBPs, is dominated by calcium-binding epidermal growth factor-like (cbEGF) domains (Figure 1). These are interspersed by transforming growth factor-β binding protein-like (TB) domains throughout the polypeptides. Related domains, the hybrid (hyb) domains, which have sequences similar to both the TB and cbEGF domains, are found near the N termini of these molecules, with two being found in each of the fibrillins (Corson et al., 2004; Pereira et al., 1993; Zhang et al., 1994), and one in each of the LTBPs (Gibson et al., 1995; Giltay et al., 1997; Kanzaki et al., 1990; Yin et al., 1995). The fibrillin-1 hyb domains have recently been shown to play a role in the structural integrity of the microfibrils (Mellody et al., 2006) and to be involved in interactions with other extracellular matrix proteins (El-Hallous et al., 2007). The formation of intermolecular disulphide bonds involving a conserved extra cysteine in domain hyb1 has been suggested as an early part of the microfibril assembly process (Reinhardt et al., 2000). Although there are significant sequence similarities between the hyb and TB domains, there have been no high-resolution data available for the structure of hyb domains or for their interactions with adjacent domains. This information is required to provide constraints on models for the arrangement of fibrillin in the microfibrils and to understand the spatial arrangement of cell-matrix molecules that interact with these domain types.

Here we present the X-ray crystal structure of a cbEGF9-hyb2-cbEGF10 triple domain fragment from fibrillin-1, providing the first high-resolution structure of a hyb domain found in the fibrillin/LTBP protein family. We show that these highly disulphide-bonded domains are structurally related to both the TB and EGF domains, as suggested by previous sequence data (Corson et al., 1993; Pereira et al., 1993), with an N-terminal region containing a hydrophobic core consisting of a conserved tryptophan as in the TB domains (Lee et al., 2004; Yuan et al., 1997), and the C-terminal region containing a disulphide-stabilized β sheet similar to some EGF domains (Knott et al., 1996). Measurements of Ca^2+^ binding by both of the fibrillin-1 hyb-cbEGF domain pairs show that the cbEGF domains in this context bind with high affinity, with K_d_ values in the low nanomolar range. As in the case of TB-cbEGF domain pairs, this is due to interdomain hydrophobic interactions stabilizing the Ca^2+^ binding pocket of the cbEGF domain. These data can be used to extend our knowledge of fibrillin structure and provide new constraints for models of microfibril organization.

## Results

### cbEGF9-hyb2-cbEGF10 and cbEGF22-TB4-cbEGF23 Constructs of Fibrillin-1 Have a Similar Shape

To characterize fully the architecture and interdomain interactions of the hyb-2 domain and its adjacent cbEGF domains, a crystal structure of the fibrillin-1 construct cbEGF9-hyb2-cbEGF10 (Figure 1) was determined. Crystals were grown in the presence of 20 mM CaCl_2_ in order to saturate the N-terminal cbEGF9 Ca^2+^ binding site, which is likely to have a relatively low affinity for Ca^2+^ based on previous work (McGettrick et al., 2000; Smallridge et al., 1999, 2003). Crystals diffracted X-rays to high resolution (1.7 Å for synchrotron data and 2.2 Å for in-house data used for sulfur single wavelength anomalous dispersion [SAD]) and belonged to space group P2_1_2_1_2_1_ (Table 1). Initial phases were determined experimentally by a combination of sulfur SAD and anomalous signals from bound Ca^2+^ (E.D. Lowe, S.A. Jensen, P.A. Handford, V. Achard, and E.F. Garman, unpublished data).

**Figure 2:**
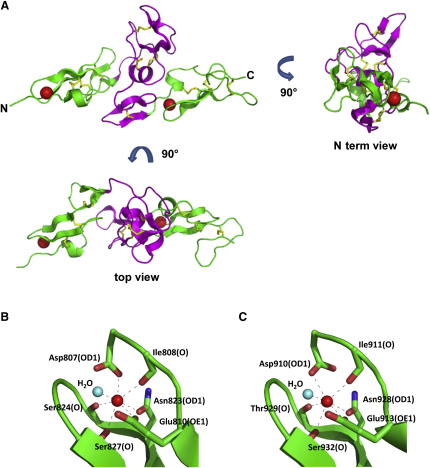
Crystal Structure of Fibrillin-1 cbEGF9-hyb2-cbEGF10 Crystals were grown in the presence of 20 mM CaCl_2_ to saturate the Ca^2+^ binding sites of both cbEGF domains. The cbEGF domains are shown in green and the hyb2 domain in magenta. Ca^2+^ bound to the cbEGF domains is represented by red spheres and disulphide bonds are shown in yellow. (A) Views either down the molecule (N terminus view) or from above the molecule (top view) show the overall linear arrangement of the domains. Ca^2+^ coordination in cbEGF9 (B) and cbEGF10 (C) was through conserved backbone and side-chain interactions as seen in previously determined structures (Abbott et al., 2007; Cordle et al., 2008; Lee et al., 2004; Rao et al., 1995).

The overall shape of cbEGF9-hyb2-cbEGF10 (Figure 2A) resembled the tetragonal pyramidal shape previously seen in the fibrillin-1 cbEGF22-TB4-cbEGF23 structure (Lee et al., 2004), with slightly smaller dimensions of approximately 6.5 × 3.5 × 2.5 nm. Like the TB4 fragment, extensive interdomain contacts were observed between cbEGF9 and hyb2, and between hyb2 and cbEGF10. As expected, both of the cbEGF domains in the structure were found to contain bound Ca^2+^. In cbEGF9, Ca^2+^ was coordinated by three side-chain oxygens (Asp807, Glu810, and Asn823), three main-chain carbonyls (Ile808, Ser824, and Ser827), and a single water molecule (Figure 2B). Coordination of the Ca^2+^ in cbEGF10 was through interactions with three side-chain oxygens (Asp910, Glu913, and Asn928), three main-chain carbonyls (Ile911, Thr929, and Ser932), and a single water molecule (Figure 2C). In both cases the Ca^2+^ ligands, from highly conserved residues involved in Ca^2+^ binding in previously determined structures (Abbott et al., 2007; Cordle et al., 2008; Lee et al., 2004; Rao et al., 1995) were arranged with approximate pentagonal bipyramidal geometry.

**Figure 3:**
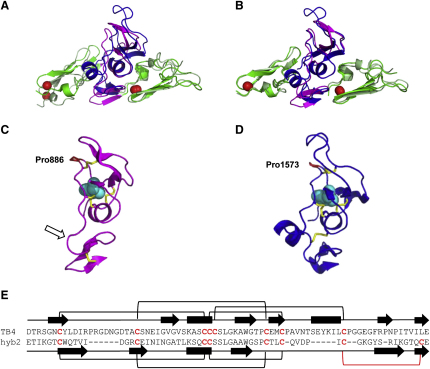
Comparison of Hyb2 and TB4 Domain Structures (A) Superposition of cbEGF9-hyb2-cbEGF10 onto the previously determined structure of fibrillin-1 fragment cbEGF22-TB4_cbEGF23 (Lee et al., 2004) showing the overall similarity of the structures. (B) Superpostion of individual domains of cbEGF9-hyb2-cbEGF10 onto the corresponding regions of cbEGF22-TB4-cbEGF23 indicated that differences in the structures were largely due to small differences seen in the interdomain packing interactions of the different constructs. (C and D) Domain hyb2 (C) has secondary structure elements similar to those seen in TB4 (D) (Lee et al., 2004). A conserved tryptophan (cyan spheres in the 3D models) is involved in forming the hydrophobic core of each domain. The arrow in (C) highlights the loss of an α helix in hyb2 relative to the corresponding position in TB4. (E) Comparison of the TB4 and hyb2 domain sequences with the disulphide bond pairings in each indicated. The rearrangement of disulphides in hyb2 compared with TB4 results in the stabilization of a C-terminal β sheet in the hyb2 domain (red bracket).

A superposition of the cbEGF9-hyb2-cbEGF10 structure onto cbEGF22-TB4-cbEGF23 highlighted the high degree of similarity between the structures (Figure 3A). The relatively high root-mean-square deviation (rmsd) value derived from the superposition (2.96 Å) was likely due to small differences in domain packing. Superpositions of the individual domains cbEGF9, hyb2 and cbEGF10 structure onto the corresponding domains in cbEGF22-TB4-cbEGF23 (Figure 3B) showed higher degrees of similarity (rmsd = 0.829, 1.844, and 1.668, respectively), consistent with the conserved nature of the cbEGF fold and similarities between TB and hyb domain sequences.

### Domain Hyb2 Has a TB Domain-Like Fold with a Rearrangement of Disulphides

The hyb2 domain structure is similar to the previously determined TB domain structures (Lack et al., 2003; Lee et al., 2004; Yuan et al., 1997) (Figures 3C and 3D). Both domain types have a central α helix, containing the Cys-Cys-Cys motif of the TB domain and Cys-Cys dipeptide of the hyb domain (Figures 3C and 3D). Similar stretches of β sheet are also seen in both domain types, with a three- or four-stranded section near the N-terminal half of the domain and a two-stranded section of β sheet near the C terminus. A conserved *cis*-proline (Pro886) is also seen in the hyb2 domain in the position corresponding to the *cis*-proline observed previously in the TB4 structure (Pro1573) (Lee et al., 2004). The major secondary structural difference between the two domain types is the absence of a second stretch of α helix in the hybrid domain (indicated by an arrow in Figure 3C).

In both the TB and hyb domains, the first two of the cysteines (C3 and C4) in the central α helix form disulphide bonds with cysteines toward the N-terminal part of the domain near the hydrophobic core. In the TB domains, a third disulphide is formed between C5 in the central α helix and C8, which is positioned between the second α helix and the final two strands of β sheet. The hyb2 structure shows a rearrangement of disulphides toward the C-terminal part of the domain so that the cysteine corresponding to C8 in the TB domain forms a disulphide bond with the second to last residue of the domain. This is likely to stabilize the C-terminal β sheet of the hyb domain and changes the arrangement of disulphides from C1-3, 2-6, 4-7, 5-8 in the TB domains to C1-3, 2-5, 4-6, 7-8 in the hyb domain (Figure 3E).

### Hyb2 Forms Extensive Contacts with Adjacent cbEGF Domains in the Presence of Ca^2+^

**Figure 4:**
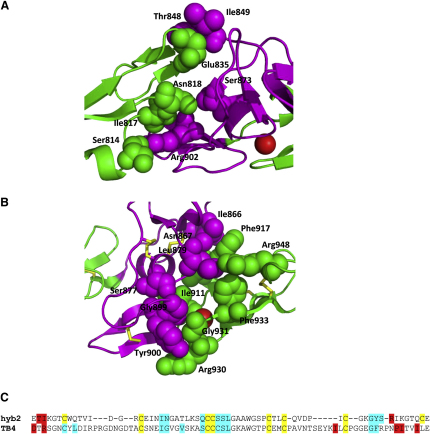
Residues Involved in the Formation of Interdomain Interfaces (A and B) Residues involved in interdomain contacts were identified using the program Contact in the CCP4 program suite (CCP4, 1994), with a distance limit of 4 Å. Hyb domain residues at the cbEGF9-hyb2 interface (A) or hyb2-cbEGF10 interface (B) are indicated by magenta spheres and interacting cbEGF residues are indicated by green spheres. (C) An alignment of the hyb2 and TB4 domains shows similarities of the regions involved in interactions with either the N-terminal (red) or C-terminal (cyan) cbEGFs. Domains were aligned using Clustal W 2.0 (Larkin et al., 2007), and cysteines are highlighted in yellow.

An understanding of how adjacent domains in fibrillin interact is required to provide important constraints on how individual fibrillin molecules can be arranged within the microfibril. Previous studies have shown that Ca^2+^ plays an important role in the formation of interfaces within cbEGF-cbEGF and TB-cbEGF pairs, and confers a rigidity to these structures (Jensen et al., 2005; Kettle et al., 1999; Knott et al., 1996; Smallridge et al., 1999; Whiteman et al., 1998). To examine the interactions of domain hyb2, points of contact at the interdomain interfaces and buried surface areas of cbEGF9-hyb2-cbEGF10 were determined from the crystal structure using the programs Contact and AreaIMol in the CCP4 program suite (CCP4, 1994). Using a 4.0 Å maximum contact distance, residues in hyb2 found to interact with parts of cbEGF9 included Thr848, Ile849, Ser873, and Arg902 (Figure 4A). At the other side of the hyb2 domain, residues Ile866, Asn867, Ser877, Leu879, Gly899, and Tyr900 were found to be involved in interdomain contacts with cbEGF10 residues (Figure 4B). A comparison of the residues in hyb2 and TB4 that interact with the adjacent cbEGF domains (Figure 4C) shows that there are conserved patches of sequence that are involved in packing with either the N-terminal or C-terminal adjacent domain.

At the cbEGF9-hyb2 interface, the buried surface area was 451.1 Å^2^, averaged between the two surfaces, and the corresponding value for the hyb2-cbEGF10 interface was 552.3 Å^2^. The formation of interdomain interfaces is predicted to result in approximately 20% of the available surface area of the individual domains being buried. In comparison, the TB4-cbEGF23 interface measured by Lee et al. (2004) was found to bury a surface area of 670 Å^2^, which is comparable to the 700 Å^2^ typical of an antibody-antigen interface (Colman, 1988). cbEGF-cbEGF domain interfaces are typically less extensive, with buried average surface areas of 220.15 Å^2^ at the cbEGF12-cbEGF13 interface and 183.55 Å^2^ at the cbEGF32-33 interface as measured using AreaIMol and previously determined structures (Downing et al., 1996; Smallridge et al., 2003). Thus, the interactions mediated by hyb and TB domains are likely to have a significant impact on the overall stability and shape of the fibrillin-1 molecule.

### The “Hybrid” Nature of the Hyb Domain Sequence Is Reflected in its Structure

**Figure 5:**
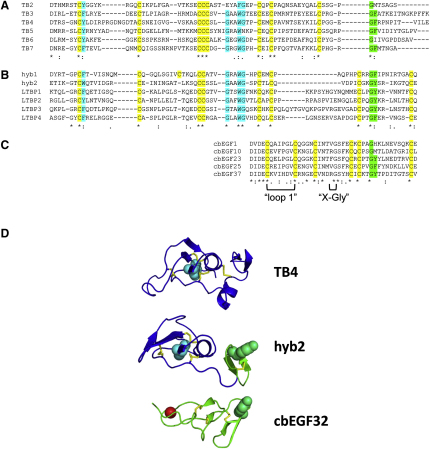
Alignments of TB, Hybrid, and cbEGF Domains (A–C) TB domains from fibrillin-1 (A), hybrid domains from fibrillin-1 (hyb1 and hyb2) and LTBPs 1-4 (B), and the cbEGF domains found in fibrillin-1 hyb-cbEGF domain pairs and high Ca^2+^ affinity fibrillin-1 TB-cbEGF pairs (C) were aligned using Clustal W 2.0 (Larkin et al., 2007). Cysteine residues are highlighted in yellow. The positions of the “loop 1” and “X-Gly” interdomain packing sites of the cbEGFs are indicated. Regions that show a high degree of sequence conservation between TB and hyb domains are highlighted in cyan. The Gly-Phe/Tyr dipeptide found at the C-terminal end of the domains, which has previously been shown to be involved in interdomain interactions in TB-cbEGF pairs (Jensen et al., 2005; Lee et al., 2004) and cbEGF-cbEGF pairs (Knott et al., 1996; Smallridge et al., 2003), is highlighted in green. (D) Comparison of the hyb2 domain structure with the structures of cbEGF (green) and TB (blue) domains mirrors the sequence alignment data and confirms the “hybrid” nature of the domain. The conserved Trp at the hydrophobic core of the TB domain is shown as cyan spheres, and the conserved aromatic found at the C-terminal packing site of the cbEGF is shown as green spheres.

Early sequencing data for *FBN1* cDNA suggested that fibrillin-1 had unique hybrid domains with properties that were similar to both the TB and EGF domains (Corson et al., 1993; Pereira et al., 1993). An alignment of various TB and cbEGF domains from fibrillin-1 with the hybrid domains of fibrillin-1 and the LTBPs (Figures 5A–5C) shows these similarities. The fibrillin and LTBP hybrid domain sequences share several conserved features including the positions of 8 cysteine residues, a conserved aromatic residue at the N terminus of the domain, a central Gly-x-x-Trp-Gly sequence, and a Gly-Phe/Tyr dipeptide at the C terminus. A comparison of the hybrid domains with the TB domains of fibrillin-1 shows that they share similar features at their N-terminal ends, with the exception of the Cys-Cys-Cys sequence in the TB domain being replaced with a Cys-Cys dipeptide in the hyb domains.

Conservation of the N-terminal aromatic and a central Gly-x-x-Trp-Gly sequence that forms a hydrophobic core in TB domains (Lack et al., 2003; Lee et al., 2004; Yuan et al., 1997) suggests structural similarities between these domain types (Figure 5D). The C-terminal end of the hyb domain resembles the sequence found at the C-terminal end of the cbEGF sequences of fibrillin-1, with closely related sequence lengths and conservation of a Gly-Phe/Tyr dipeptide that is involved in interdomain interactions in cbEGF tandem repeats. These structural observations are consistent with a mechanism for the evolution of the hyb domain from a TB-cbEGF pair.

### Hyb-cbEGF Domain Pairs Have a High Ca^2+^ Affinity

A previous study of the Ca^2+^ binding properties of TB-cbEGF pairs showed that Ca^2+^ affinity was strongly influenced by the presence of a hydrophobic interdomain interface. The parts of the cbEGF domain found experimentally to be involved in this interaction were the X-Gly interdomain packing site in the central β sheet of the domain, and the “loop 1” sequence (Figure 5C) found between C1 and C2 of the cbEGF domain (Jensen et al., 2005). A comparison of the cbEGF domains in the fibrillin-1 hyb1-cbEGF1 and hyb2-cbEGF10 pairs with those found in high Ca^2+^ affinity domain pairs TB4-cbEGF23, TB5-cbEGF25, and TB7-cbEGF37 shows similarities in these regions (Figure 5C). The loop 1 sequences of cbEGF1 (CQAIPGLC) and cbEGF10 (CEVFPGVC) have similar hydrophobic character to the loop 1 sequence of cbEGF23 (CQELPGLC), which is involved in hydrophobic interactions with domain TB4 and makes a significant contribution to the high Ca^2+^ affinity of the TB4-cbEGF23 domain pair. The presence of a Gly-Phe/Tyr dipeptide at the C-terminal end of the hyb domains, in a position similar to the conserved Gly-Phe/Tyr interdomain packing site found in the N-terminal domain of cbEGF-cbEGF pairs, suggested another potential site of hyb-cbEGF interdomain packing. We therefore predicted that the fibrillin-1 hyb-cbEGF domains would be sites of high Ca^2+^ affinity based on our previous TB-cbEGF data.

Assays of the hyb2-cbEGF10 pair with the chromophoric Ca^2+^ chelator Quin-2 (Table 2; see Figure S1 available online) showed that this domain pair bound with very high affinity, with a K_d_ value of 14.7 ± 3.7 nM (mean ± standard deviation [SD], n = 4). In addition, the Ca^2+^ affinity of a cbEGF9-hyb2-cbEGF10 construct (Table 2; Figure S1) was measured using Quin-2 to examine the effect of having an extra domain at the N terminus of the hyb domain. The measured K_d_ value for this construct, 12.2 ± 4.0 nM (mean ± SD, n = 5), suggested that the addition of a cbEGF to the N terminus of the hyb2 domain had no significant effect on the Ca^2+^ affinity of cbEGF10, and that the high Ca^2+^ affinity of this construct is due entirely to hyb2-cbEGF10 interfacial interactions.

**Figure 6:**
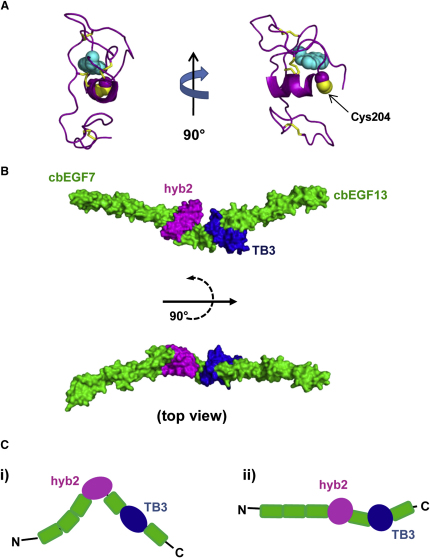
Models of Other Fibrillin-1 Regions (A) A model of domain hyb1, using the coordinates of hyb2 from the cbEGF9-hyb2-cbEGF10 structure, shows that Cys204 is likely to occur at an exposed position at the N terminus of the domain's central α helix. (B) Molecular modeling data suggest that the region from cbEGF7 to cbEGF13 is generally linear, with domains hyb2 and TB3 inverted with respect to each other. Models were created using Modeler software (Eswar et al., 2007) and the coordinates of the structures determined for fibrillin-1 fragments cbEGF12-cbEGF13 (Smallridge et al., 2003), cbEGF32-33 (Downing et al., 1996), and cbEGF22-TB4-cbEGF23 (Lee et al., 2004). (C) An earlier interpretation of the structure of the cbEGF7-cbEGF13 region (i), based on SAXS data (Baldock et al., 2006), suggested a V-shaped bend in the region of the hyb domain. High-resolution studies of hyb and TB interactions with adjacent domains (ii) suggest a more linear organization, highlighting the influence of high-resolution constraints on models to explain microfibril length and extensibility.

To test whether this was a general feature of the hyb-cbEGF pairs, Ca^2+^ binding by the hyb1-cbEGF pair of fibrillin-1 was studied. Chelation competition assays of the native hyb1-cbEGF1 domain pair were not possible due to the insolubility of this fragment. Not unexpectedly, this insolubility was found to be due to the presence of a free thiol, as determined by assays with Ellman's reagent (data not shown), which became reactive when the protein was dissolved in buffers at physiological pH. Ca^2+^ binding was therefore measured for a hyb1-cbEGF1 construct containing a Cys204Ser substitution, which removed the free thiol previously shown to be involved in intermolecular interactions (Reinhardt et al., 2000). Assays of hyb1-cbEGF1(Cys204Ser) with Quin-2 (Table 2; Figure S1) showed that this construct had a very high Ca^2+^ affinity, with a K_d_ value of 8.7 ± 3.5 nM (mean ± SD, n = 4). Molecular modeling of hyb1, based on the hyb2 structure, shows that the extra cysteine of hyb1 is likely to be positioned at the N terminus of the central α helix of the domain in a solvent-exposed position (Figure 6A).

NMR analysis of the hyb2-cbEGF10 domain pair, using 2D nuclear Overhauser enhancement spectroscopy (NOESY) and heteronuclear single quantum coherence (HSQC) spectra of ^15^N-labeled material, and assignments based on 3D HSQC-NOESY and HSQC/total correlation spectroscopy data in the presence of 5 mM Ca^2+^ or 10 mM EDTA, showed that significant conformational changes occurred on Ca^2+^ binding in solution (data not shown; S.A.J., S.I., E.D.L., C.R., and P.A.H., unpublished data). Chemical shift changes were seen for both cbEGF and hyb domain residues, indicating that interface formation in the hyb2-cbEGF10 pair is Ca^2+^-dependent as seen previously in TB-cbEGF domain pairs (Jensen et al., 2005).

### Molecular Modeling of Domains cbEGF7-cbEGF13 Suggests a Near-Linear Arrangement

The structure presented here indicates that, under physiological Ca^2+^ concentrations (∼1.5 mM free Ca^2+^), the region around hyb2 would be near linear with N and C termini emerging at opposite ends of the fragment. Based on our current knowledge of cbEGF, TB, and hyb domain structures (Lack et al., 2003; Rao et al., 1995; Smallridge et al., 2003; Yuan et al., 1997), and of cbEGF-cbEGF (Downing et al., 1996; Knott et al., 1996), TB-cbEGF (Jensen et al., 2005; Lee et al., 2004), and hyb-cbEGF interdomain interactions, we can now produce molecular models of most of the fibrillin molecule. Using high-resolution structural data and an understanding of interactions that occur at interdomain interfaces, we propose that fibrillin monomers form largely extended structures, at least between domains cbEGF3 and cbEGF43. This is consistent with several electron microscopic studies of fibrillin monomers and recombinant fragments (Hubmacher et al., 2008; Lin et al., 2002; Reinhardt et al., 1997a). The region around domain hyb2 is of particular interest because it contains all three of these interface types and is also at the beginning of the “neonatal” region of fibrillin affected by mutations resulting in the neonatal Marfan syndrome phenotype. Based on domain structure and interface data, a model of the fibrillin-1 fragment spanning the region from cbEGF7-cbEGF13 (Figure 6B) was produced. The results suggest a linear organization of the region, with a slight twist in the region of hyb2 and TB3 in which these two domains appear inverted with respect to each other.

## Discussion

Our study has provided the first high-resolution structure and possible evolutionary origin of a hybrid domain, one of the major disulphide-rich domains of the fibrillin/LTBP superfamily. Overall the fibrillin-1 hyb2 domain has a globular shape, resembling a TB domain, and is involved in extensive interactions with its neighboring domains, cbEGF9 and cbEGF10. A mechanism for the evolution of the hybrid domain as a fusion of TB and cbEGF domains was first proposed by Pereira et al. (1993). They showed that the N-terminal sequence of hyb2, encoded by exon 21, is homologous to the sequence encoded by the upstream exon of a 2-exon TB domain and that the C-terminal part of domain hyb2, encoded by exon 22, has a similar spacing between the cysteines as the C-terminal third of a cbEGF domain. A comparison of the structures of the hyb2, TB, and cbEGF domains indicates that this similarity in sequences is conserved at the structural level, suggesting that these domains arose from the fusion of a TB-cbEGF pair (Figure 5D). The functional significance of the rearrangement of disulphides in a hyb domain compared with a TB domain is unclear. It has been proposed that the C-terminal linker region of fibrillin domain TB4 could act as a molecular spring (Lee et al., 2004), with application of a tensional force causing unfolding of the C-terminal β-hairpin that could allow the region to extend by up to 5 nm. This would not be possible in a hybrid domain, with the C-terminal β-hairpin constrained by a disulphide bond, but a different mechanism of extension might be possible in this case. An unstructured loop between C6 and C7 (^890^CQVDPIC^896^) of hyb2 separates the N-terminal TB-like region from the C-terminal cbEGF-like region. Under tension, this loop could allow the disruption of the hyb-cbEGF interface, extending the length of the structure by approximately 3.5 nm. Recoil of the system would be achieved by the reformation of the hyb-cbEGF interface, which is stabilized by the binding of Ca^2+^ binding to cbEGF10. Such dynamic behavior could facilitate the structural role of fibrillin in connective tissues that are subjected to high tensional forces.

The organization of fibrillin in the 10–12 nm diameter microfibrils is still a topic of controversy, with several models having been proposed based on a wide range of experimental techniques (Baldock et al., 2001, 2006; Knott et al., 1996; Kuo et al., 2007; Lee et al., 2004; Reinhardt et al., 1996; Smallridge et al., 2003). An understanding of the interdomain interactions within fibrillin is required for the development of any model of microfibril organization. Binding of calcium to the cbEGF domains rigidifies the structure of tandem cbEGF repeats and has been shown to determine the shape of fibrillin and protect it against proteolytic cleavage (Reinhardt et al., 1997a, 1997b). High-resolution structures of cbEGF domain pairs have shown that calcium binding to cbEGF domains stabilizes hydrophobic interdomain contacts (Knott et al., 1996; Smallridge et al., 2003). Solution and X-ray crystal structures of a number of TB domain-containing fragments (Lack et al., 2003; Lee et al., 2004; Yuan et al., 1997) have shown that these domains are stabilized by disulphide bonds in a C1-3, 2-6, 4-7, 5-8 arrangement, and contain a conserved aromatic residue that forms the hydrophobic core of the domain. In fibrillin-1, extensive interactions at cbEGF-TB and TB-cbEGF interfaces have been observed in an integrin-binding fragment consisting of domains cbEGF22-TB4-cbEGF23. The high Ca^2+^ affinity of the TB4-cbEGF23 pair (K_d_ = 16 nM) in this fragment was in contrast to previous observations of a TB6-cbEGF32 fragment (K_d_ = 1.6 mM), suggesting that Ca^2+^ affinities across the molecule could vary substantially. This was found to be the case in a study of all fibrillin-1 TB-cbEGF domain pairs, where it was also shown that Ca^2+^ affinity could be correlated with the degree of interdomain interaction. Given the relatively high affinities seen for all the TB-cbEGF pairs except TB6-cbEGF32, it was suggested that these regions of fibrillin form rigidified, linear structures at Ca^2+^ concentrations found in the extracellular matrix (Jensen et al., 2005).

The overall structure of the Ca^2+^-saturated fibrillin-1 cbEGF9-hyb2-cbEGF10 fragment determined here generally resembles the cbEGF22-TB4-cbEGF23 integrin-binding fragment described previously (Lee et al., 2004), with extensive interdomain interactions that are Ca^2+^-dependent at the hyb2-cbEGF10 interface. As in the case of the TB4-cbEGF23 domain pair (Jensen et al., 2005), the Ca^2+^ affinity of hyb2-cbEGF10 was found to be much higher than would be required to reach saturation under the Ca^2+^ concentrations found in the extracellular matrix. The buried surface area at the hyb-cbEGF interface was slightly less than the 670 Å^2^ seen at the TB4-cbEGF23 interface (Lee et al., 2004). The significance of these very high Ca^2+^ affinity sites is not understood, but might be related to the proposed extensible nature of the microfibrils in dynamic connective tissues. Extensive hydrophobic interactions at hyb-cbEGF and TB-cbEGF interfaces, stabilized by Ca^2+^, could provide the driving force for recoil after the application of a tensile force. An extensive protein-binding interface between hyb1 and cbEGF1, as suggested by the high Ca^2+^ affinity observed for this domain pair, would also explain the context-dependent interactions of domain hyb1 with the fibulins seen by El-Hallous et al. (2007). Domain hyb1, when placed in the position of domain hyb2, was unable to “drive” the binding of recombinant fibrillin-1 fragments to fibulins, suggesting that a specific molecular surface formed by the interactions of hyb1 with its adjacent domains is required for these intermolecular interactions.

Structural studies on fibrillin fragments are also highly relevant to the closely related LTBPs, each of which contains one hyb domain in the context of a hyb-cbEGF-TB fragment bordered by relatively long stretches (89–148 residues) of cysteine-free sequence. LTBPs 1, 3 and 4 have been shown to bind covalently the latency-associated peptide of TGFβ through the formation of an intermolecular disulphide bond, with the interaction mediated by the rearrangement of a disulphide bond in the second TB domain of the LTBPs. The structural basis of this rearrangement has been well-characterized and is due to the presence of a two amino acid insertion between C6 and C7 of the TB domains, leading to the increased solvent accessibility of the C2-C6 disulphide bond. In the fibrillin-1 hyb2 structure, no comparable arrangement of disulphides was observed, suggesting that this domain is unlikely to be involved in disulphide rearrangements. In contrast, domain hyb1 has been shown to be involved in the formation of intermolecular disulphides as a result of the presence of a conserved 9th cysteine (Reinhardt et al., 2000). Our model of domain hyb1, based on the structure of hyb2 determined in the cbEGF9-hyb2-cbEGF10 structure, is consistent with a surface-exposed position for Cys204, near the N-terminal end of the central α helix of the domain (Figure 6A), and suggests that both TB and hyb domains contain potential protein binding surfaces distinct from their intramolecular interfaces.

A fibrillin fragment spanning from cbEGF7-cbEGF11 has recently been studied by SAXS (Baldock et al., 2006). Rigid body modeling to the SAXS data, using known cbEGF and TB domain structures, produced a V-shaped structure with the bend at the hyb domain (Figure 6C[i]), in contrast to the model based on X-ray crystallographic data (Figure 6C[ii]). In these previous studies, modeling of the hyb domain region was possible only for the N-terminal region homologous to TB domains, but without high-resolution structural data it would not have been possible to model accurately the interaction of hyb2 with the adjacent domains. The Ca^2+^-saturated cbEGF9-hyb2-cbEGF10 structure presented in our study shows that domains cbEGF9 and cbEGF10 pack against domain hyb2 to form a near-linear structure. Ca^2+^ binding data for the hyb2-cbEGF10 pair shows that an extensive interface is formed between these domains, as seen previously in TB-cbEGF pairs where specific hydrophobic contacts between domains were found to influence Ca^2+^ affinity (Jensen et al., 2005). These differences in the observed and modeled hyb domain structures highlight the importance of high-resolution data in the development of models to explain the organization of fibrillin microfibrils. Variations in the way domains pack against each other can lead to substantial differences in the predicted dimensions and shape of full-length fibrillin monomers (Figure 6C), which can have a significant impact on how these data are used to model microfibril organization. Although dissection approaches for structure determination have limitations in that studies can be time-consuming and might not detect long-range interactions involving flexible regions, the identification of interdomain interactions at atomic resolution provides an essential constraint for microfibril modeling.

In summary, the data presented here extend our knowledge of the structural properties of fibrillin-1 and provide further evidence for an extended model of microfibril organization. In addition, our data suggest a mechanism by which microfibril extensibility could be achieved that does not disrupt potential binding sites for microfibril accessory proteins.

## Experimental Procedures

### Cloning and Expression of Fibrillin Constructs

All recombinant constructs were expressed in *Escherichia coli* strain BL21 as His_6_-tagged fusion proteins using the pQE-30 expression vector (Qiagen). Constructs corresponding to protein fragments hyb1-cbEGF1 (Arg179 to Glu287), cbEGF9-cbEGF10 (Asp807 to Leu951), and hyb2-cbEGF10 (Glu847 to Leu951) were amplified from a human fibrillin-1 cDNA (Kettle et al., 2000) using *Pfu* polymerase and cloned into the expression vector pQE-30 (Knott et al., 1996). A Cys204Ser substitution was introduced into the hyb1-cbEGF1 construct, to produce hyb1-cbEGF1(C204S), by site-directed mutagenesis using an overlapping polymerase chain reaction method as described previously (Jensen et al., 2005).

Expression, in vitro refolding and purification were essentially as described (Knott et al., 1996) except in the case of hyb1-cbEGF1(C204S), for which glycerol was added to 50% (v/v) to the refolding mixture to maintain protein solubility. An additional dialysis step, against 0.1% (v/v) trifluoroacetic acid, was added before high-performance liquid chromatography (HPLC) purification of the refolding mixture in these cases to reduce the viscosity of the solution.

### Crystallization and Structure Determination

Crystallization trials were carried out using solutions of cbEGF9-hyb2-cbEGF10 prepared at 15 mg/ml in 20 mM CaCl_2_. Sparse matrix screening and optimization produced rod-shaped crystals using a reservoir solution of 100 mM Tris (pH 7.5), 200 mM NaI, and 16% PEG 3350. All crystals were washed with mother liquor supplemented with 25% (v/v) glycerol before storage in liquid nitrogen. Native X-ray diffraction data were collected at the European Synchrotron Radiation Facility (ESRF). Initial phases were determined from SAD data collected in-house using a Bruker MicroStar Smart 6000 CCD X-ray generator, with signals from both sulfur and calcium (E.D. Lowe, S.A. Jensen, P.A. Handford, V. Achard, and E.F. Garman, unpublished data). The structure was rebuilt and refined using Coot (Emsley and Cowtan, 2004) and phenix.refine (Adams et al., 2002) software.

### Chromophoric Ca^2+^ Chelator Competition Assays

Ca^2+^ dissociation constants were determined by competition with the chromophoric Ca^2+^ chelator Quin-2 as described previously (Linse et al., 1991). To minimize background levels of Ca^2+^ in protein solutions, all proteins were treated with 5 mM EDTA prior to purification by HPLC. Ca^2+^-free solutions of proteins in Ca^2+^-free buffer (5 mM Tris, pH 7.5, 150 mM NaCl) were titrated with Ca^2+^ stock buffer (5 mM Tris, pH 7.5, 150 mM NaCl, 1 mM CaCl_2_) in the presence of Quin-2. All titrations were performed with 20 – 30 μM chelator and 20 – 30 μM protein at 23 ± 2°C using a Shimadzu UV mini 1240 spectrophotometer to monitor absorbance at 263 nm. Background Ca^2+^ levels in the protein-containing samples were determined by extrapolating the initial linear region of each titration curve back to the *y*-axis. The initial Ca^2+^ concentration of each curve was adjusted so that the *y*-intercept was equivalent to that of a “chelator-only” control. The K_d_ value for Quin-2 was determined independently from chelator only control experiments; a K_d_ value of 0.35 μM for Quin-2 was obtained. Free Ca^2+^ concentration at each titration point was calculated by the Newton-Raphson method, and dissociation constants were calculated by least-squares fitting to the data, as described previously using in-house software (Jensen et al., 2005; Linse et al., 1991; Stenberg et al., 1997). Errors in the measured K_d_ values were estimated from at least four repeats of each assay.

## Figures and Tables

**Table 1:** Data Collection and Refinement Statistics

Data Collection		
X-ray source	ESRF ID23.EH1	In-house
Space group	P2_1_2_1_2_1_	P2_1_2_1_2_1_
Unit cell dimensions (a, b, c) (Å) (α, β, γ) (°)	31.30, 47.63, 89.36, 90, 90, 90	31.232, 47.258, 89.105, 90, 90, 90
Molecules in the asymmetric unit	1	1
Resolution range (outer shell) (Å)^a^	29.79-1.70 (1.79-1.70)	89.10-2.20 (2.30-2.20)
Number of reflections	15,382	12,682
Completeness (%)^a^	100.0 (100.0)	99.8 (99.0)
Multiplicity^a^	13.3 (13.7)	38.09 (17.63)
*I/*σ (*I*)^a^	24.3 (7.6)	27.24 (5.66)
R_pim_	0.026 (0.132)	
R_sigma_^a^^b^		0.0302 (0.1879)

Refinement Statistics		

R_working_ (%)	18.51	
R_free_ (%)	22.12	
Solvent content	42.07	
Average B factors (Å^2^)	23.78	
Average B factors for Ca^2+^ (Å^2^)	17.31	
Rmsd from ideal values		
Bond lengths (Å)	0.006	
Bond angles (°)	1.027	

a Values in parentheses correspond to the highest-resolution shell.

b R_sigma_ =$\sum \left[\sigma \left(F_{0}^{2}\right)\right]/\sum \left[F_{0}^{2}\right]$

**Table 2:** Calcium Dissociation Constants Determined by Competition with Quin-2

Fibrillin-1 Fragment	K_d_ (nM)^a^
hyb1-cbEGF1(C204S)	8.7 ± 3.5, n = 4
hyb2-cbEGF10	14.7 ± 3.7, n = 5
cbEGF9-hyb2-cbEGF10	12.2 ± 4.0, n = 4

a Values shown are mean ± SD.

## Accession Numbers

Atomic coordinates and structure factors have been deposited in the Protein Data Bank under the ID code 2w86.

## Supplemental Data

Document S1. One Figure
