# Supplementary material for: Structure and Interdomain Interactions of a Hybrid Domain: A Disulphide-Rich Module of the Fibrillin/LTBP Superfamily of Matrix Proteins
Source: Structure. 2009 May 13;17(5-3):759–68. doi: 10.1016/j.str.2009.03.014 (PMC2724076; doi:10.1016/j.str.2009.03.014)
Supplement: Document S1. One Figure [file mmc1.pdf]

Supplemental Data

Structure and Interdomain Interactions of a Hybrid Domain: A Disulphide-Rich Module of the Fibrillin/LTBP Superfamily of Matrix Proteins

Sacha A. Jensen, Sarah Iqbal, Edward D. Lowe, Christina Redfield, and Penny A. Handford

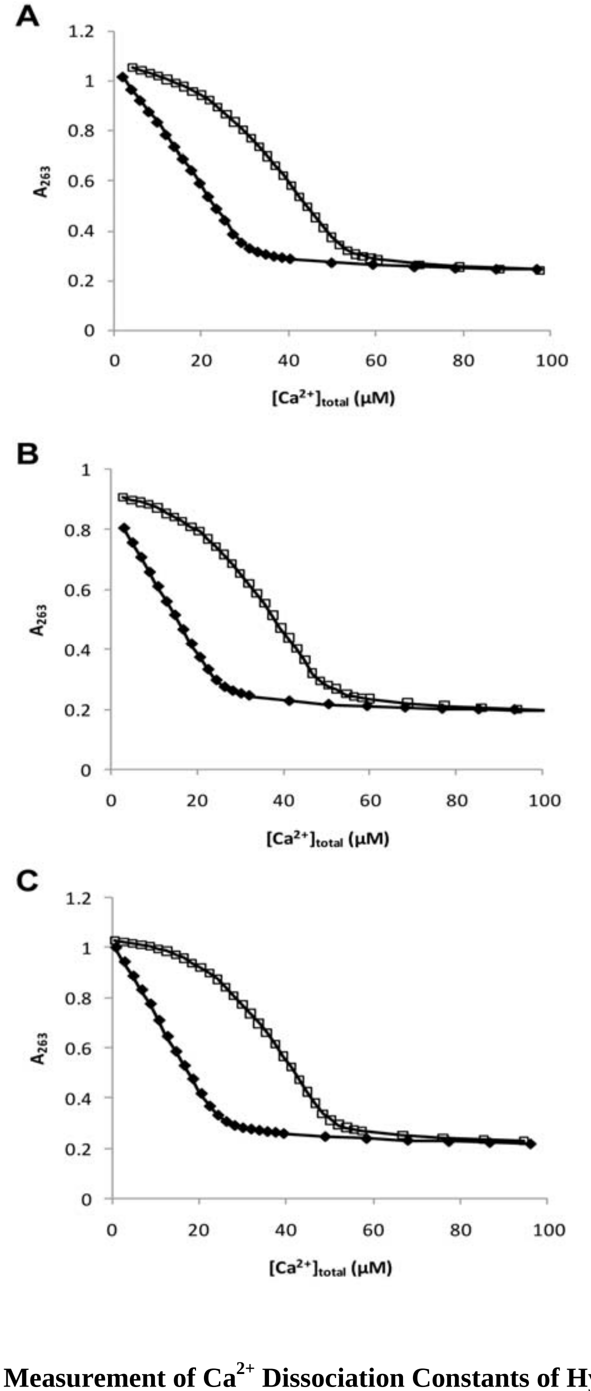

Figure S1. Measurement of Ca^{2+} Dissociation Constants of Hyb Domain Constructs using the Chromophoric Chelator Quin-2

Constructs were dissolved in Chelex-treated Ca^{2+}-free buffer (5 mM Tris, pH 7.5 and 150 mM NaCl) to a concentration of 25-30 μM and titrated with Ca^{2+} in the presence of 25-30 μM Quin-2. Curves shown are the results of titrations of chelator alone (diamonds) or chelator with protein (open squares) and are representative of the data used to determine K_d values for each construct.

(A) Titration of 29.2 μM Quin-2 with 29.5 μM hyb2-cbEGF10.

(B) Titration of 24.9 μM Quin-2 with 25.0 μM cbEGF9-hyb2-cbEGF10.

(C) Titration of 28.2 μM Quin-2 with 29.8 μM hyb1-cbEGF1(C204S).
